# Type of acute periprosthetic joint infection may not affect failure of debridement, antibiotics, and implant retention after total knee arthroplasty

**DOI:** 10.5194/jbji-10-225-2025

**Published:** 2025-07-23

**Authors:** Andrew J. Frear, Michael F. Shannon, Shaan Sadhwani, Anthony O. Kamson, Clair Smith, Charity G. Patterson, Victoria R. Wong, Frank Johannes Plate, Kenneth L. Urish

**Affiliations:** 1 School of Medicine, University of Pittsburgh, Pittsburgh, PA 15203, USA; 2 Department of Orthopaedic Surgery, UPMC Central PA, Harrisburg, PA 17109, USA; 3 School of Health and Rehabilitation Sciences, University of Pittsburgh, Pittsburgh, PA 15219, USA; 4 Department of Orthopaedic Surgery, University of Pittsburgh, Pittsburgh, PA 15232, USA; 5 Arthritis and Arthroplasty Design Group, University of Pittsburgh, Pittsburgh, PA 15219, USA; 6 Department of Bioengineering, University of Pittsburgh, Pittsburgh, PA 15261, USA; 7 Clinical and Translational Science Institute, University of Pittsburgh, Pittsburgh, PA 15213, USA

## Abstract

**Introduction:** In periprosthetic joint infection (PJI) following total knee arthroplasty (TKA), debridement, antibiotics, and implant retention (DAIR) is a common procedure with a high rate of failure. Timing of infection can be used to stratify acute PJI into acute postoperative, intermediate, and hematogenous infections. Potential differences in prognosis between classifications remain unclear. This investigation assessed the current overall failure of DAIR procedures, compared DAIR failure between three types of acute PJI, and analyzed DAIR outcomes in an “optimal” cohort of patients with a minimal number of medical comorbidities.

**Methods:** This retrospective study compared 122 patients with acute TKA PJI who underwent DAIR between 2016 and 2022. Categorization was based on timing between index TKA and PJI diagnosis, with < 6 weeks termed postoperative ( n= 43), 6 weeks to 1 year termed intermediate ( n= 19), and > 1 year termed hematogenous ( n= 60). The primary outcome was DAIR failure, defined as reoperation for PJI. Subgroup analysis was performed after removing patients with high-risk comorbidities.

**Results:** The overall failure rate was 42 %; 78.4 % of failures occurred within 1 year. No significant differences in the failure rate were found between PJI types at any time point. At 1 year, 58 % postoperative, 58 % intermediate, and 77 % hematogenous cases remained failure-free ( p= 0.09). Failure rates of 45 % postoperative, 44 % intermediate, and 36 % hematogenous cases were seen in the optimal cohort, without significant differences.

**Conclusions:** DAIR failure for acute TKA PJI is high. Although no differences in failure rates were observed based on the PJI type, DAIR failure trended lower for the hematogenous group in this study. Outcomes for DAIR appear similar regardless of the PJI type in optimal patients.

## 1 Introduction

Periprosthetic joint infection (PJI), the most common cause of revision in total knee arthroplasty (TKA) (Bozic et al., 2010), is associated with high morbidity, mortality, and cost (Drain et al., 2022; Sadhwani et al., 2024). Debridement, antibiotics, and implant retention (DAIR) is the preferred treatment for acute PJI due to this method's advantageous simplicity, bone stock preservation, decreased anesthesia duration, lower blood loss, lower fracture risk, and fewer surgeries (Longo et al., 2024; Zhang et al., 2022). Notable failure risk is among the disadvantages of DAIR; previous retrospective literature has described failure rates of between 33 % and 64 % (Urish et al., 2018; Zhu et al., 2021; Iza et al., 2019) with an overall rate of approximately 50 % (Kurtz et al., 2012; Urish et al., 2018).

Acute PJI is defined by the International Consensus Meeting (ICM) to have occurred within 4 weeks of the first presenting symptom (Parvizi et al., 2014). Acute PJI can be further differentiated based upon the time of occurrence following TKA. Acute postoperative PJI manifests within 6 weeks following the initial surgery, likely related to intraoperative contamination. PJI developing over 1 year following index TKA is defined as acute hematogenous PJI and sources from circulatory dissemination (Kuiper et al., 2014). Given their distinct pathogeneses, these two categories may carry different prognoses (Lima et al., 2013). However, few studies have compared outcomes following DAIR between acute postoperative and acute hematogenous infection (Chotanaphuti et al., 2019). Furthermore, there is a paucity of data regarding the “intermediate” time frame between acute postoperative and acute hematogenous PJI. This window of 6 weeks to 1 year after TKA has been designated by some as the “delayed period” (Tsikopoulos and Meroni, 2023; Weinstein et al., 2023). These infections may represent late presentation of acute postoperative PJI, early development of acute hematogenous PJI, or another etiology entirely (Chotanaphuti et al., 2019).

The purpose of this study was to compare failure rates after DAIR between these three classifications of acute PJI. Specific aims included the following: (1) assessing the current overall failure of DAIR procedures, (2) comparing DAIR outcomes in treating acute postoperative vs. acute hematogenous PJI, (3) evaluating DAIR failure when including a subset of patients with acute intermediate PJI, and (4) analyzing DAIR outcomes across PJI types in an “optimal” cohort of patients without high-risk comorbidities.

## 2 Methods

An Institutional Review Board (IRB)-approved, retrospective study of patients between 1 January 2016 and 1 July 2022 with acute PJI who underwent a DAIR procedure was performed. Patient demographics and clinical data were obtained via electronic medical record (EMR) information from multiple hospitals within a regional health system, including academic facilities and community hospitals, serving both urban and rural populations.

### 2.1 Definitions

In this study, acute PJI was characterized by diagnosis less than 4 weeks after the first presenting symptom. Chronic PJI was defined by more than 4 weeks of symptoms at the time of diagnosis. All patients with acute PJI were further stratified using established temporal criteria (Gristina and Kolkin, 1983; Tsukayama et al., 1996; Zimmerli et al., 2004). Acute PJI diagnosed within 6 weeks of primary TKA was defined as postoperative PJI. Acute PJI diagnosed more than 1 year after primary TKA was defined as hematogenous PJI. The definition of intermediate PJI was attributed to cases diagnosed between 6 weeks and 1 year following primary TKA. The primary outcome of “DAIR failure” was defined as a repeated surgical procedure for recurrent PJI on the ipsilateral knee joint, corresponding with 2019 Musculoskeletal Infection Society (MSIS) Outcome Reporting Tool tiers 3B or 3D (Fillingham et al., 2019). Manipulation under anesthesia for arthrofibrosis was not considered a repeated surgical procedure. “Time to failure” was defined as the time between first incision of DAIR and the subsequent surgery. Mortality was determined via documentation in the EMR database. Follow-up was determined as an outpatient visit documented in the EMR. All facilities within the regional health system utilize a unified electronic record system, ensuring the continuity of documentation.

### 2.2 Population

Patients were initially identified through electronic medical records using the International Classification for PJI (ICD codes: 996.66, T84.53XA, and T84.54XA). As an additional check, surgical billing procedures associated with acute PJI were also reviewed. These patient lists were combined, duplicates were removed, and the population was screened for inclusion and exclusion criteria. Patients who received primary TKA at outside institutions were included as long as appropriate documentation of the date and location was identified in the EMR. Criteria for inclusion in this study were diagnosis with acute PJI (symptom duration less than 4 weeks and management with debridement, antibiotics, and implant retention with polyethylene liner exchange. Adherence to the 2013 MSIS criteria was confirmed for all patients to ensure consistency in defining PJI (Parvizi et al., 2013). For all patients, a symptom-free period was observed between primary TKA and the first presenting symptom.

**Figure 1:**
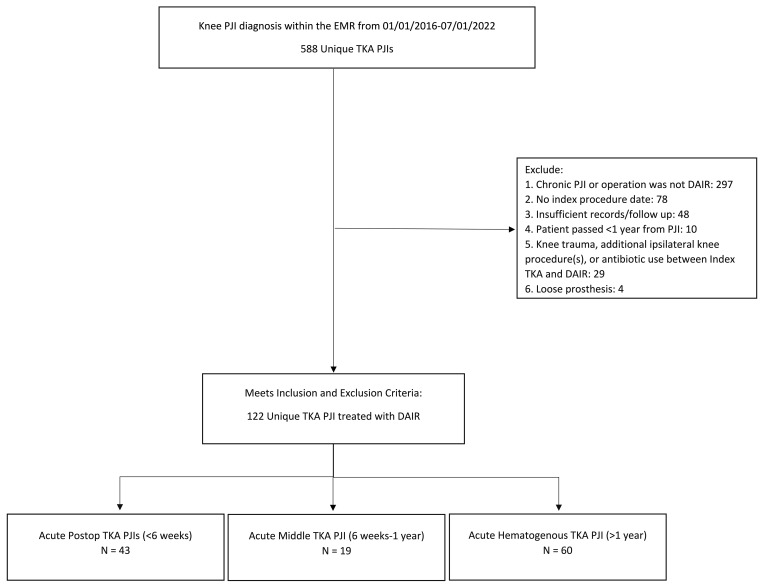
The flow diagram demonstrates the method for patient inclusion, exclusion, and categorization by type of acute PJI.

From the original chart extraction, 588 patients were identified. Study personnel (Andrew J. Frear, Michael F. Shannon, Shaan Sadhwani, Anthony O. Kamson, and Victoria R. Wong) manually reviewed records to collect clinical information, including relevant laboratory values, antibiotic usage, and microbiology results. The Charlson comorbidity index (CCMI) was calculated for each patient using a standardized formula (Charlson et al., 1994). Criteria for exclusion from the study included the following: chronic PJI or operative treatment other than DAIR with polyethylene exchange ( n= 297); no documentation of index procedure date ( n= 78); knee trauma, additional surgical procedures, or antibiotic use between index TKA and DAIR ( n= 29); and loose prosthesis ( n= 4). A prior study identified a significant breakpoint in DAIR failure rates at 1 year, with 2 years considered to be an appropriate follow-up duration (Chao et al., 2024a). Therefore, patients with less than a 2-year follow-up and no documentation of failure were also excluded ( n= 58). After the exclusion criteria were applied, 122 patients remained. Patients were subsequently stratified into acute postoperative, intermediate, and hematogenous subgroups according to the criteria above (Fig. 1). A board-certified, fellowship-trained arthroplasty surgeon reviewed all cases to confirm that each patient (1) received a DAIR procedure, (2) was assigned to the correct subgroup, (3) met the criteria for acute PJI, (4) met the MSIS 2013 criteria, and (5) fit the inclusion and exclusion criteria.

For secondary analysis, patients were further stratified into two groups based on the presence of high-risk comorbidities for PJI treatment failure. Comorbid conditions were determined through ICD-10 codes obtained from the EMR information. Risk factors collated from previous studies (Shohat et al., 2020; Tohidi et al., 2019; Xu et al., 2020) included the following: an age of greater than 80 years; BMI > 45 kg m^−2^; immunocompromised status; venous thromboembolism (VTE) history; uncontrolled diabetes (A1C > 9.0); PJI in another joint; sepsis; and active alcohol, tobacco, or drug use disorder. A total of 59 patients displayed no high-risk comorbidities for PJI and, therefore, met the criteria for inclusion in this secondary analysis.

### 2.3 Description of treatment

In all cases, the attending surgeon led the patient's diagnosis, surgery, and treatment. Individuals were diagnosed using the preoperative and intraoperative MSIS 2013 criteria. Tests used to analyze PJI included the following: C-reactive protein; erythrocyte sedimentation rate; synovial fluid analysis; cultures of fluid, tissues, and sonication of prosthesis; histologic analysis; and observations. For all patients, DAIR was the primary surgical treatment for PJI. The polyethylene insert was exchanged in all cases. Postoperatively, input was sought from an infectious disease specialist to guide and manage antimicrobial therapy. Antibiotic regimens followed Infectious Diseases Society of America (IDSA) guidelines for PJI management. As the analysis of records was retrospective, specific operative technique and postoperative antibiotic treatment varied between subjects.

### 2.4 Data analysis

Categorical variables were summarized with percentages and frequencies. Continuous variables were described with means and standard deviations. Categorical variables were compared between groups with a chi-square or Fisher exact test. Continuous variables were compared with analysis of variance (ANOVA) or a Fisher exact test; post hoc comparisons were made between groups using an independent samples t test or Fisher exact test. Post hoc testing did not control for multiplicity. All tests were two-sided. Survival plots of time until failure with 95 % confidence limits were constructed using the Kaplan–Meier method; differences between groups were tested with the log-rank test. Patients lost to follow-up after 2 years were appropriately censored. There was no preplanned sample size or power analysis conducted for this study, as this was a retrospective observational cohort. The significance level was set at 0.05. All analyses were performed in SAS version 9.4 (SAS Institute Incorporated, Cary, NC, USA).

## 3 Results

### 3.1 Demographics and description of study participants

Of the 122 patients meeting inclusion criteria, 43 were classified as acute postoperative, 19 as acute intermediate, and 60 as acute hematogenous PJI. The average age for the overall sample was 62.9 ± 10.7 years, the average BMI was 33.3 ± 7.1 kg m^−2^, and 50 % of patients were female. No significant differences were detected between groups with respect to sex ( p= 0.06) or BMI ( p= 0.23). The acute postoperative cohort was younger ( p= 0.0002) with lower ASA ( p= 0.01) and CCMI ( p= 0.01) scores. The proportion of diabetes mellitus was highest in the hematogenous group ( p= 0.004). Mean follow-up duration was also recorded, with the greatest average follow-up period in the acute postoperative group at 1460 d (4 years) (Table 1).

**Table 1:** Patient demographics.

Demographics	Total	Groups			p value	p value	p value	p value
	( n= 122)				ANOVA	1 vs. 2	1 vs. 3	2 vs. 3
		Postoperative	Intermediate	Hematogenous				
		( n= 43)	( n=19 )	( n=60 )				
Age (in years),	68.1	62.9	70.9	70.9	**0.0002**	**0.004**	< **0.0001**	0.98
mean (SD)	(10.7)	(9.0)	(11.1)	(10.4)				
BMI (in kg m^−2^),	33.3	34.8	32.2	32.6	0.23	–	–	–
mean (SD)	(7.1)	(7.0)	(6.5)	(7.4)				
Length of follow-up	1276.0	1460.2	1056.8	1213.4	**0.04**	**0.04**	0.06	0.31
(in days), mean (SD)	(646.7)	(717.4)	(587.6)	(584.5)				
Male sex,	61	18	14	29	0.06	–	–	–
N (%)	(50)	(42)	(74)	(48)				
Diabetes,	28	3	4	21	**0.004**	0.19	**0.001**	0.25
N (%)	(23)	(7)	(21)	(35)				
Immunosuppressed,	7	2	1	4	1.00	–	–	–
N (%)	(6)	(5)	(5)	(7)				
ASA class,	2.9	2.7	3.1	3.0	**0.01**	**0.03**	**0.01**	0.67
mean (SD)	(0.5)	(0.5)	(0.5)	(0.5)				
CCMI score,	3.5	2.8	3.7	3.8	**0.01**	0.06	**0.003**	0.85
mean (SD)	(1.7)	(1.6)	(1.8)	(1.6)				

Groups: postoperative – group 1; intermediate – group 2; hematogenous – group 3. Abbreviations: SD – standard deviation; BMI – body mass index. Significant p values are shown using bold font.

Sample PJI characteristics showed mean erythrocyte sedimentation rate (ESR) of 72.5 ± 33.3 mm h^−1^, a mean C-reactive protein (CRP) value of 19.8 ± 29.5 mg L^−1^, and an average antibiotic duration of 254.5 ± 383.1 d. No significant differences were detected between groups with respect to ESR ( p= 0.97) or CRP ( p= 0.75). The average duration of antibiotic therapy in the hematogenous group was 149.2 d longer than the postoperative group and 227.1 d longer than the intermediate group ( p= 0.003). Of all cases, 39 % were caused by *Staphylococcus aureus*. A lower proportion of infection with *S. aureus* was seen in the hematogenous cohort relative to both comparators ( p= 0.001 and p= 0.04). PJI caused by *Streptococcus* spp. was significantly less common in the acute postoperative cohort ( p= 0.034). No significant differences in the proportion of infections caused by coagulase-negative staphylococci (CoNS) or polymicrobial etiology were observed (Table 2). Other implicated species included *Escherichia coli*, *Citrobacter* spp., *Pseudomonas aeruginosa*, *Enterococcus faecalis*, *Haemophilus influenzae*, *Pasteurella multocida*, and *Serratia marcescens*. Culture-negative infection was seen in 17 % of all cases without significant differences between groups ( p= 0.70).

**Table 2:** PJI characteristics.

PJI	Total	Groups			p value	p value	p value	p value
characteristic	( n= 122)				ANOVA	1 vs. 2	1 vs. 3	2 vs. 3
		Postoperative	Intermediate	Hematogenous				
		( n= 43)	( n= 19)	( n= 60)				
ESR (in mm h^−1^),	72.5	73.3	73.1	71.7	0.97	–	–	–
mean (SD) ( n= 99)	(33.3)	(32.0)	(36.2)	(33.6)				
CRP (in mg L^−1^),	19.8	21.9	22.6	17.7	0.75	–	–	–
mean (SD) ( n= 101)	(29.5)	(47.3)	(28.9)	(10.3)				
Length of antibiotics (in days),	254.5	194.0	116.1	343.2	**0.03**	0.17	**0.04**	**0.002**
mean (SD) ( n= 121)	(383.1)	(226.1)	(142.9)	(493.2)				
Patients on antibiotic								
suppression, n (%)								
( n= 121)								
3 months	63 (52.1)	25 (58.1)	5 (26.3)	33 (55.9)	0.052	–	–	–
6 months	53 (43.8)	17 (39.5)	4 (21.1)	32 (54.2)	**0.034**	0.26	0.16	**0.017**
12 months	32 (26.4)	8 (18.6)	3 (15.8)	21 (35.6)	0.10	–	–	–
24 months	8 (6.6)	1 (2.3)	0 (0)	7 (11.9)	0.10	–	–	–
*S. aureus*,	47	24	9	14	**0.003**	0.54	**0.001**	**0.04**
N (%)	(39 %)	(56 %)	(47 %)	(23 %)				
Coagulase-negative	8	4	1	3	0.077	–	–	–
staphylococci, N (%)	(6.5 %)	(9.3 %)	(5.3 %)	(5.0 %)				
*Streptococcus* spp.,	26	1	6	19	**0.034**	**0.022**	**0.019**	0.21
N (%)	(21.3 %)	(2.3 %)	(31.6 %)	(31.7 %)				
Polymicrobial,	9	4	1	4	0.89	–	–	–
N (%)	(7 %)	(9 %)	(6 %)	(7 %)				
Culture-negative,	21	8	2	11	0.70	–	–	–
N (%)	(17 %)	(19 %)	(11 %)	(18 %)				
Other microbial	11	7	0	4	0.07	–	–	–
species^*^	(9 %)	(11.7 %)	(0 %)	(0.3 %)				

Groups: postoperative – group 1; intermediate – group 2; hematogenous – group 3. Abbreviations: SD – standard deviation; ESR – erythrocyte sedimentation rate; CRP – C-reactive protein; CCMI – Charlson comorbidity index; ASA – physical status classification system of the American Society of Anesthesiologists. ^*^ Other microbial species included *Citrobacter*, *Enterococcus*, *Haemophilus*, *Pseudomonas*, *Pasteurella*, and *Serratia* spp. Significant p values are shown using bold font.

### 3.2 Primary outcome – DAIR failure as reoperation

**Figure 2:**
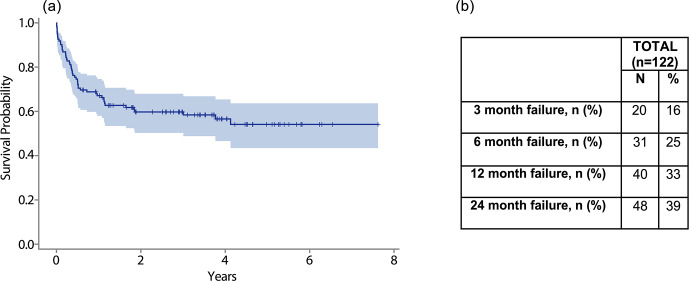
**(a)** The graph shows a Kaplan–Meier survival curve for overall survival after DAIR. **(b)** The table shows overall failure rates at 3 months, 6 months, 1 year, and 2 years.

The overall estimated cumulative failure rate for this cohort was 42 %. Of these failures, 78 % occurred within 1 year. Just over one-third (39 %) of failures occurred within the first 3 months following the procedure (Fig. 2a). The failure rate was 25 % at 6 months and 39 % at 2 years following DAIR (Fig. 2b).

### 3.3 Reoperation – acute postoperative PJI and acute hematogenous PJI

**Figure 3:**
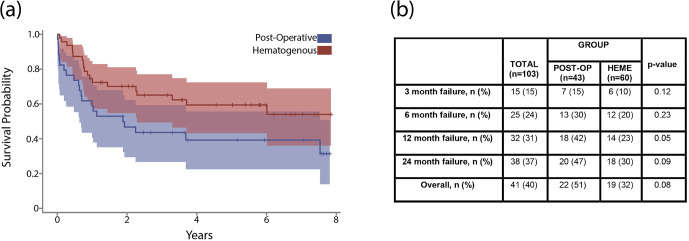
**(a)** The graph shows a Kaplan–Meier survival curve after DAIR for acute postoperative and acute hematogenous PJI. **(b)** The table shows failure rates after DAIR for acute postoperative and acute hematogenous PJI at 3 months, 6 months, 1 year, and 2 years.

At final follow-up, estimated cumulative failure rates for the acute postoperative and hematogenous PJI groups were 51 % and 32 %, respectively ( p= 0.09). A Kaplan–Meier analysis with 95 % confidence intervals demonstrated no significant difference in time until failure between groups (Fig. 3a). Chi-square testing revealed no significant differences in cumulative failure between groups at the 3-month, 6-month, 12-month, and 24-month time points (Fig. 3b). Cumulative failure rates at 1 year were 42 % and 23 % for acute postoperative and acute hematogenous PJI, respectively ( p= 0.05). Two-year failure rates of 47 % for postoperative and 30 % for hematogenous PJI were recorded ( p= 0.09).

### 3.4 Reoperation – acute postoperative, acute intermediate, and acute hematogenous PJI

**Figure 4:**
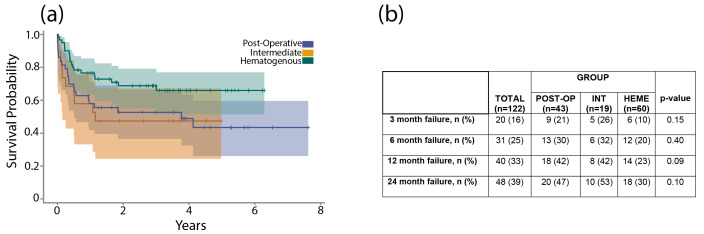
**(a)** The graph shows a Kaplan–Meier survival curve after DAIR for acute postoperative, acute hematogenous, and acute intermediate PJI. **(b)** The table shows failure rates after DAIR for acute postoperative, acute hematogenous, and acute intermediate PJI at 3 months, 6 months, 1 year, and 2 years.

The acute intermediate PJI group demonstrated a 1-year failure rate of 42 % and a 2-year failure rate of 53 %. A Kaplan–Meier analysis revealed no significant difference in time to failure between the three acute PJI classifications (Fig. 4a). Chi-square testing revealed no significant differences in failure rate at 3 months ( p= 0.15), 6 months ( p= 0.40), 1 year ( p= 0.09), or 2 years ( p= 0.10) (Fig. 4b).

### 3.5 Secondary analysis – time until failure after DAIR in an “optimal” PJI patient cohort

**Figure 5:**
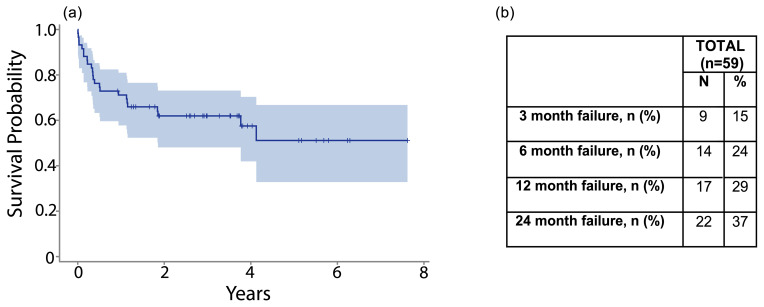
**(a)** The graph shows a Kaplan–Meier survival curve after DAIR in the optimized cohort. **(b)** The table shows failure rates after DAIR in the optimized cohort at 3 months, 6 months, 1 year, and 2 years.

**Figure 6:**
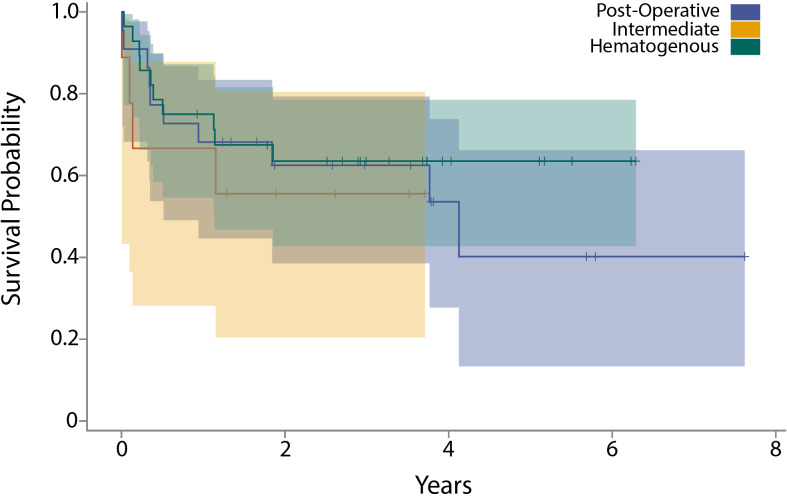
The graph shows a Kaplan–Meier survival curve after DAIR for acute postoperative, acute hematogenous, and acute intermediate PJI in the optimized cohort.

Patients with at least one high-risk comorbidity associated with DAIR failure ( n= 63, 52 %) were excluded from secondary analysis in order to create an “optimal” PJI cohort (Table 3). Common comorbidities included the following: age ≥ 80 years (16 %); presentation with sepsis (11 %); and active alcohol, tobacco, or drug abuse at the time of DAIR (22 %). Distribution of comorbidities varied between PJI groups, but no significant differences were observed in the total number of patients with at least one high-risk comorbidity ( p= 0.90). After exclusion, 59 patients remained in the “optimal” cohort. The overall 2-year failure rate in this group was 37 % (Fig. 5a and b). When these patients were separated by PJI definitions (22 postoperative, 9 intermediate, 28 hematogenous), the time until failure between groups was not significantly different ( p= 0.76) (Fig. 6).

**Table 3:** High-risk PJI comorbidities by group. No significant difference was seen in the number of patients with at least one comorbidity between groups.

Comorbidity	Total ( n= 122)	Groups			p value
		Postop. ( n= 43)	Int. ( n= 19)	Heme. ( n= 60)	
One or more high-risk comorbidities, n (%)	61 (50)	21 (49)	8 (42)	32 (53)	0.90
80 + years of age, n (%)	19 (16)	3 (7)	4 (21)	12 (20)	
PJI in another joint, n (%)	5 (4)	1 (2)	0 (0)	4 (7)	
Sepsis, n (%)	13 (11)	2 (5)	2 (11)	9 (15)	
Uncontrolled diabetes, n (%)	5 (4)	1 (2)	0 (0)	4 (7)	
BMI 45 + (in kg m^−2^), n (%)	6 (5)	3 (7)	1 (5)	2 (3)	
Active alcohol, tobacco, or drug abuse, n (%)	27 (22)	15 (35)	4 (21)	8 (13)	
Chronic immunosuppressant use, n (%)	6 (5)	2 (5)	1 (5)	3 (5)	
Physiologically immunocompromised, n (%)	2 (2)	0 (0)	0 (0)	2 (3)	
Venous thromboembolism, n (%)	4 (3)	0 (0)	1 (5)	3 (5)	

Groups: Postop. – postoperative; Int. – intermediate; Heme. – hematogenous. Abbreviations: PJI – periprosthetic joint infection; BMI – body mass index.

## 4 Discussion

While DAIR has a high failure rate (Toh et al., 2021), its advantages include retaining primary implants, lower surgical morbidity, and a short operative duration compared to two-stage exchange. However, DAIR outcomes relative to PJI timing after index TKA require further exploration to appropriately anticipate prognosis. Prior work has evaluated DAIR for early ( < 1 year) and hematogenous ( > 1 year) designations (Zhu et al., 2021), However, study comparing DAIR failure for acute postoperative ( < 6 weeks), intermediate (6 weeks to 1 year), and hematogenous ( > 1 year) classifications is limited. This retrospective study analyzed DAIR outcomes in relation to these three types of acute PJI, finding similarly high failure rates regardless of timing. While a trend toward higher failure for postoperative and intermediate PJI was observed, larger sample sizes and additional study is likely necessary.

Overall, we observed a high rate of 2-year failure (39 %) and a pattern of rapid failure within 1 year, comparable to previous large studies on DAIR for acute PJI (Urish et al., 2018; Zhang et al., 2022; Zhu et al., 2021). When stratified by type of acute infection, differences in overall DAIR failure were observed between postoperative (51 %) and hematogenous PJI (32 %). Likewise, failure by 1 year was 42 % for postoperative PJI vs. 23 % for hematogenous PJI. Although statistical significance was not achieved, these disparities are noteworthy and may reflect meaningful underlying prognostic differences. Further investigation is needed, as methodologies and results are inconsistent among prior studies. One retrospective study of 230 PJIs analyzed DAIR effectiveness for early ( < 1 year) and late ( > 1 year) PJI and found a significantly lower rate in the late-PJI cohort (38 % vs. 64 %) (Zhu et al., 2021). Other studies suggest similar results between classifications, provided the infection is acute (Lowik et al., 2020; Iza et al., 2019). A systematic review of DAIR for hematogenous PJI, defined as PJI > 3 months from index TKA, presented similar failure rates to other published literature on postoperative DAIR (Balato et al., 2022). In a retrospective review of TKA PJI treated with DAIR, Sherrell et al. (2011) found no significant difference between DAIRs < 4 weeks and > 4 weeks from the index procedure.

In this study, infections diagnosed < 6 weeks from primary TKA were designated as postoperative, those > 1 year from primary TKA were considered hematogenous, and the intervening period was defined as “intermediate”. However, classifications of acute PJI vary across the literature (Masters et al., 2019). Tsukayama et al. (1996) originally established “postoperative” to be within 4 weeks after index surgery and “hematogenous” to be after an asymptomatic period. Subsequently, others have used “postoperative” to characterize infections within either 6 weeks (Xu et al., 2019) or 3 months after TKA (Zimmerli et al., 1998; Martinez-Pastor et al., 2009; Aboltins et al., 2011). Given this heterogeneity, the definition, pathogenesis, and prognosis for “intermediate” PJI remain contentious. Our data did not show a significant difference in DAIR failure for intermediate PJI compared to other types of acute infection. Despite this, a high degree of overlap was seen in the 95 % confidence limits of survival curves for the postoperative and intermediate groups. This study may have been underpowered to prove that greater prognostic similarity exists between these two types of PJI relative to hematogenous PJI. Further investigation, including prospective studies with large sample sizes, is needed to evaluate this potential relationship.

The relationship between microbiology and DAIR failure has been well described. Numerous authors have associated *Staphylococcus aureus*, polymicrobial, and culture-negative infections with poor outcomes (Chen et al., 2021; Bernaus et al., 2022; Iza et al., 2019; Urish et al., 2018). However, literature describing microbiology in the period between postoperative and hematogenous infection is sparse. Nearly 40 % of our sample developed PJI due to *S. aureus*, supporting the prevalence of this species in early PJI reported in other studies (Longo et al., 2024). Of note, *S. aureus* was isolated less frequently in the hematogenous cohort compared with both comparator groups. This greater incidence in the postoperative and intermediate PJI groups may be attributable to postoperative contamination, given the ubiquitous presence of *Staphylococcus* in skin flora (Anderson and Kaye, 2009). Here, greater prevalence of *S. aureus* did not translate into a significantly higher failure rate for either group. In fact, the similar failure rate in the hematogenous cohort despite the lower incidence of this high-risk pathogen could suggest that outcomes for hematogenous PJI are truly worse overall. This hypothesis warrants further investigation, such as a targeted analysis of hematogenous PJI with and without *S. aureus*. In contrast, *Streptococcus* spp. (i.e., β -hemolytic *Streptococci*) was isolated less frequently in the acute postoperative group. This corroborates a recent study by Weinstein et al. (2023), who reported that *Streptococcus* was more common in both intermediate and “late” infections, equivalent to our definition of hematogenous PJI. Interestingly, their study noted a significantly greater proportion of Gram-negative pathogens in acute postoperative PJI, whereas our findings demonstrated equipoise across groups. Further studies involving greater pathogen diversity may help to fully establish how individual causal pathogens influence outcomes between types of acute PJI.

Although the timeline is not fully understood, in vitro study has suggested that antibiotic-tolerant biofilm can form within days (Lebeaux et al., 2013), which may impact treatment resistance. It has been hypothesized that intermediate PJI stems from a delayed response to postoperative infection with subtle symptoms, prolonging the opportunity for biofilm development. Moreover, pathogens identified across all three PJI groups such as *Staphylococcus, Streptococcus*, and Gram-negative bacilli can produce biofilm (Zimmerli and Sendi, 2017). However, the precise role that biofilm may play in shaping outcomes for postoperative, intermediate, and hematogenous PJI is beyond the scope of this retrospective study. Future prospective investigations should incorporate the quantification of biofilm across PJI types, bridging the intersection between acute and chronic infection.

The secondary aim of this study was to assess the risk of DAIR failure in an optimal population after removing high-risk patients. Prior studies have commented on comorbidities and lab values associated with an increased risk of PJI complications (Shohat et al., 2020; Tohidi et al., 2019; Xu et al., 2020). By reviewing similar literature, we collated a list of high-risk comorbidities. Despite removing these patients, no significant difference was seen in DAIR outcomes between the three classifications. The original trend seen in the total cohort was no longer observed with optimization. Our data suggest that the type of acute PJI does not significantly influence clinical outcomes following DAIR in patients with limited risk factors, but it could have an impact for higher-risk patients.

Our study is not without limitations. Given the retrospective observational design, details of care, such as indications for surgery and the duration between diagnosis and DAIR procedure, varied between patients. To account for this variability, the MSIS 2013 criteria were used to standardize the definition of PJI. The hematogenous cohort had longer durations of antibiotic suppression, which was not a subject of investigation in our study. Further investigation at our selected time points showed minimal significant variance in antibiotic suppression. While IDSA guidelines were followed for antibiotic selection, the consensus on suppressive antibiotics is constantly evolving, and variation was seen among subjects (Chao et al., 2024b). As guidelines progressively advance, continued surveillance on the role of antibiotic suppression after acute DAIR is needed to optimize therapeutic strategies across all types of acute PJI. Although only acute infections with a symptom duration of less than 4 weeks were included, the exact duration between symptom onset and DAIR did not factor into the analysis. As data were accumulated retrospectively using notes from the EMR, some records outside our health system were unavailable, precluding the documentation of outcomes. Additionally, defining failure via additional procedures for PJI is a controversial limitation. Other markers for ineffective treatment, such as patient-reported outcomes, were not included in our failure characterization. Furthermore, after 2 years of follow-up, the three subgroups had a varied duration of median follow-up, potentially contributing to differences in long-term outcomes. These outcomes are valuable in assessing overall treatment success and may provide more comprehensive understanding of patient outcomes.

Overall, these findings should be interpreted with caution, and the true impact of PJI type on failure risk requires further exploration to determine clinical utility. In the future, larger and more robust studies should be considered to more conclusively determine how the timing of acute PJI development impacts patient outcomes.

## 5 Conclusions

Although reported rates vary across the literature, the DAIR failure rate remains high. In this study, we observed no statistically significant difference in DAIR outcomes in all three classifications of acute PJI (postoperative, intermediate, and hematogenous infections), although there was a trend towards higher failure rates in postoperative and intermediate PJI. In patients with limited risk factors, there was no difference between groups and no observed trend. Continued evaluation and further prospective studies are warranted.

## Data Availability

The data used to conduct this study are patient data from our institution. All data were collected and stored securely within database software that is used by our institution. Among the data are items that are identifying information for patients; for this reason, data are not publicly available.
